# Haplotype Analysis of β-Thalassaemia Major and Carriers with Filipino β°-Deletion in Sabah, Malaysia

**DOI:** 10.21315/mjms2018.25.4.6

**Published:** 2018-08-30

**Authors:** Lai Kuan Teh, George Elizabeth, Mei I Lai, Lily Wong, Patimah Ismail

**Affiliations:** 1 Department of Biomedical Science, Faculty of Sciences, Universiti Tunku Abdul Rahman, 39100 Kampar, Perak, Malaysia; 2 Department of Pathology, Faculty of Medicine and Health Sciences, Universiti Putra Malaysia, 43400 Serdang, Selangor, Malaysia; 3 Assunta Hospital, Jalan Templer, Petaling Jaya, Selangor, Malaysia; 4 Department of Medicine, Hospital Queen Elizabeth, 88586 Kota Kinabalu, Sabah, Malaysia; 5 Department of Biomedical Sciences, Faculty of Medicine and Health Sciences, Universiti Putra Malaysia, 43400 Serdang, Selangor, Malaysia

**Keywords:** haplotypes, β-thalassaemia major, β-thalassaemia carrier, Filipino β°-deletion, Sabah, Malaysia

## Abstract

**Objective:**

The Filipino β°-deletion has been reported as a unique mutation in East Malaysia with a severe phenotype due to the complete absence of β-globin chain synthesis. In this study, the haplotype patterns of the β-globin gene cluster were used to relate the human genetic variation to this specific β-thalassaemia mutation.

**Methods:**

The 376 study subjects included 219 β-thalassaemia major (β-TM) patients with homozygous Filipino β°-deletion and 157 carriers with heterozygous Filipino β°-deletion from 10 government hospitals in different regions of Sabah. Genomic DNA was isolated from whole blood using silica membrane based DNA purification protocol. Polymerase chain reaction restriction fragment length polymorphism analysis (PCR-RFLP) was conducted on five markers within the β-globin gene cluster to construct the haplotype patterns.

**Results:**

Four haplotypes (Haplotype I–IV) were identified with Haplotype I as the predominant haplotype with the highest frequency of 0.98, followed by Haplotype II, III and Haplotype IV with 0.02. Haplotype I was strongly linked with the Filipino β°-deletion among the indigenous population.

**Conclusion:**

Haplotype I as the predominant haplotype suggests the patients with the Filipino β°-deletion in Sabah have a similar origin.

## Introduction

Beta thalassaemia (β-thalassaemia) is a haemoglobin disorder caused by either the reduction or complete absence of β-globin chain production. The clinical variability is based on inherited mutations. There are over 250 β-thalassaemia mutations recorded worldwide with up to 90% caused by point mutations (1). Only a small proportion is caused by genomic deletions in the β-globin gene cluster such as the Filipino β°-deletion (1). The Filipino β°-deletion [NG_000007.3:g.66258_184734del118477] was first reported in a Filipino family (2). In later, Filipino β°-deletion was also found in 45.8% of the β-globin mutant alleles in Filipinos in Taiwan (3), 86.9% of transfusion dependent β-TM patients in Sabah (4), 16 out of 125 members of the Kadazandusun ethnic group (5), 19 out of 20 β-TM families (6) and 20 Dusun children from the indigenous communities in Sabah, East Malaysia (7).

The Filipino β°-deletion is the largest deletion seen among the reported β-thalassaemia mutations. It leads to a more severe β°-thalassaemia phenotype with a complete absence of β-globin chain expression. This deletion was found to be 118 kb (8) or 116.4 kb (9) with the 5’ deletion breakpoint at the position −4279 relative to the mRNA capsite of the β-globin gene, and the 3’ breakpoint in a new L1-like sequence extending to 113 kb apart from β-globin gene and six olfactory receptor (OR) genes which consist of four functional OR genes and two OR pseudogenes including one γ-globin enhancer located at OR52A1 (2, 8, 10). Filipino β°-deletions involving deletion of the β-globin gene promoter region without deletion of the δ-globin gene will demonstrate raised HbA_2_ levels (11).

The population of Sabah in East Malaysia is predominantly indigenous with their own distinct set of β-thalassaemia mutations (4). The Filipino β°-deletion has been reported as the predominant mutation in Sabah, East Malaysia (4). The haplotype pattern for the β-globin gene is informative in determining genetic diversity diversity in a specific population based on frequencies and number of haplotypes. The increase of heterozygosity in a population may be attributed to an evolutionary process such as selection or genetic drift (12, 13). In addition, haplotype patterns are strongly associated with specific β-thalassaemia mutations and the origin of the population (13, 14). This can be seen in sickle cell disease with five independent haplotypes consisting of the Senegal (SEN) haplotype in Atlantic West Africa; Arab-Indian (ARB) haplotype in Iran, the Indian subcontinent and Eastern Arabian Peninsula; Cameroon (CAM) haplotype along the west coast of Africa; Benin (BEN) haplotype at Midwestern Africa; and the Bantu haplotype in South Central and Eastern Africa (12, 15, 16, 17). The objective of this study was to conduct a haplotyping analysis of the β-globin cluster to relate human genetic variation to a specific β-thalassaemia mutation, the Filipino β°-deletion.

## Materials and Methods

### Sample Collection

Ethical approval was obtained from the Medical Ethics Committee of the Faculty of Medicine and Health Sciences, Universiti Putra Malaysia [UPM/FPSK/PADS/T7-MJKEtikaPerF01 (JPAT_JUL(10)06)] and the Ministry of Health Medical Research Ethics Committee (MREC) [NMRR-10-850-7075] prior to commencement of the study. The study was conducted in accordance with the Declaration of Helsinki. An information sheet was provided and written informed consent was obtained from study participants prior to blood sample collection.

A total of 376 study subjects, consisting of 219 β-TM patients with homozygous Filipino β°-deletion and 157 β-thalassaemia carriers with heterozygous Filipino β°-deletion were recruited from 10 government hospitals in various regions of Sabah, East Malaysia. The hospitals were Hospital Likas (also known as *Hospital Wanita dan Kanak-kanak Sabah*), Queen Elizabeth Hospital, Hospital Kota Marudu, Hospital Pitas, Hospital Kudat, Dutches of Kent Hospital, Hospital Keningau, Hospital Tambunan, Hospital Lahad Datu and Hospital Ranau. Hospital Likas and Hospital Queen Elizabeth provided over 50% of the study subjects.

Of the 219 β-TM patients with homozygous Filipino β°-deletion, 84.5% [185 of 219] were of the indigenous population: Kadazandusun [137 of 219], Rungus [20 of 219], Murut [10 of 219], Sungai [9 of 219], Bajau [4 of 219] and Bruneian [5 out of 219 with 4 Brunei-Malay and 1 Brunei-Kedayan]. Two were mixed ethnicity (Kadazandusun/Chinese [2 of 219]). The ethnicities for the remaining 32 β-TM patients [32 of 219] were not reported.

For the 157 β-thalassaemia carriers with heterozygous Filipino β°-deletion, 47.1% [74 of 157] were indigenous with populations of Kadazandusun [47 of 157], Rungus [12 of 157], Sungai [9 of 157], Sino-Kadazan [2 of 157], Murut [2 of 157], Bisaya [1 of 157] and Bajau [1 of 157]. One was Jawa [1 of 157]. The ethnicities for the remaining 82 β-thalassaemia carriers [82 of 157] were not reported.

### DNA Isolation

Three milliliters of venous blood were collected in ethylenediaminetetraacetic acid (EDTA) vacutainers. Genomic DNA was extracted from leukocytes in peripheral whole blood samples by using a QIAamp DNA midi kit (Qiagen GmbH, Hilden, Germany). The quality and quantity of the extracted genomic DNA was determined using a Nanodrop 1000 Spectrophotometer (Thermo Scientific, Thermo Fisher Scientific Inc., Wilmington, DE, USA).

### Beta Globin Gene Cluster Haplotyping

**Figure 1:**
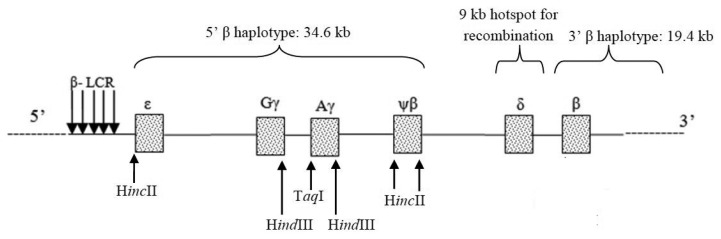
β-globin gene haplotyping. Schematic diagram demonstrates the location of the RFLP markers on the β-globin gene cluster

The beta globin (β-globin) gene cluster spans a region of 70 kb and consists of five expressed genes (ε, ^A^γ^, G^γ, δ, β) and one pseudogene (ψβ) with a 9.1 kb region recombination “hotspot” between the 5’ and 3’ β-globin gene cluster. This recombination hotspot divides the haplotypes into 5’β and 3’β-haplotypes (13, 18). This study focused on the 5’β-haplotype in subjects with the Filipino β°-deletion, an extensive deletion of the β-globin gene, including the entire region of the 3’β-globin gene cluster. The 5’β-haplotype has been widely used to investigate the evolutionary relationships between human populations (13, 18). Five restriction fragment length polymorphism (RFLP) markers within the β-globin gene complex (5’ε*-*H*inc*II, ^G^γ*-*H*ind*III, ^A^γ*-*H*ind*III, 5’ψβ*-*H*inc*II and 3’ψβ*-*H*inc*II) were included in this study to construct the haplotype patterns within the five polymorphic restriction sites (Figure 1).

Five oligonucleotide primer pairs were used for PCR amplification overlapping the five polymorphic restriction sites (Table 1) (19–22). Reactions were carried out in a 25 μL final volume consisting of 1× PCR buffer, 200 μM of each dNTP, 1.5 to 3.0 mM MgCl_2_, 0.125 to 0.05 units of Taq polymerase, 0.2 to 0.4 μM of forward and reverse primers and 100 ng of DNA templates. PCR amplification was carried out using a Takara PCR thermal cycler (Takara Bio Inc., Otsu, Shiga; Applied Biosystem Veriti^TM^ Thermal cycler, Applera Corporation, USA).

Amplified fragments were subsequently digested with five restriction enzymes. Fifteen microlitres of each amplified PCR product were digested with 10 units of Fast Digest restriction enzymes in Fast Digest Green buffers (Fermentas Life Sciences, Waltham, USA) and incubated at 37 °C for 10–25 min. Digested fragments in the Fast Digest green buffer were loaded directly to the ethidium bromide stained agarose gel and run for electrophoresis at 10 volts/cm for 30 min. The RFLP marker sites were scored by the loss or gain of restriction enzyme recognition sites. The PCR products cut by the restriction enzymes constitute a positive (+), and the absence of a cut was indicated as a negative (−).

### Analysis of Beta Globin Gene Cluster Haplotyping

The RFLP data was analysed using SNPStats from the Catalan Institute of Oncology for allele and genotype frequencies and haplotype frequency estimation (23). Inference of haplotypes from the genotype data for the RFLP markers was computed using SNPStats. Haplotype frequency estimation was carried out. Nucleotide C and G [C: presence of recognition site (+); G: absence of recognition site (−)] were used to represent binary signs for all RFLP markers for haplotype inference. The nucleotide symbols were swapped with the original (+) and (−) signs. Haplotype frequency was estimated by implementation of the Expectation Maximisation (EM) algorithm to find maximum likelihood (23).

## Results

Haplotype frequency estimation was computed using SNPStats based on the EM algorithm for a total of 752 5’β-alleles from 376 subjects. Amplification of the target site in the 5’ε-region gave a 760 bp before digestion with the *Hin*cII restriction enzyme. The absence of a recognition site (−/−) showed only 760 bp amplicon; the presence of a recognition site at one allele (+/−) showed three digested fragments (760, 446 and 314 bp) while the presence of a recognition site at both alleles (+/+) showed two digested fragments (446 and 314 bp).

Amplification of the target site in the ^G^γ-region gave an amplicon at 328 bp before digestion with the *Hind*III restriction enzyme. The absence of a recognition site at both alleles (−/−) only showed an amplicon at 328 bp; the presence of a recognition site at one allele (+/−) showed three amplicons (328, 237 and 91 bp) while the presence of recognition sites at both alleles (+/+) showed two amplicons sized 237 and 91 bp. The allele and genotype frequencies for each marker among the 376 Filipino β°-deletion study subjects are depicted in Table 2. As presented in Table 2, +/+ was the predominant genotype for 5’ε-*Hin*cII and ^G^γ-*Hin*dIII, indicating the presence of recognition sites at both alleles.

The target site in the ^A^γ-region was amplified at 761 bp. After digestion with the restriction enzyme *Hin*dIII, one amplicon at 761 bp was shown with the absence of recognition sites at both alleles (−/−); three amplicons at 761, 436 and 325 bp were shown with the presence of a recognition site at one allele (+/−) while two amplicons at 435 and 325 bp were shown with the presence of recognition sites at both alleles (+/+).

The target site in the 5’ψβ-region was amplified at 794 bp. After digestion with the restriction enzyme *Hin*cII, one amplicon at 794 bp was shown in the absence of recognition sites at both alleles (−/−); three amplicons at 794, 690 and 104 bp were shown with the presence of a recognition site at one allele (+/−) while two amplicons at 690 and 104 bp were shown with the presence of recognition sites at both alleles (+/+).

The target site in the 3’ψβ-region was amplified at 620 bp. After digestion with the restriction enzyme *Hinc*II, one amplicon at 620 bp was shown in the absence of recognition sites at both alleles (−/−); three amplicons at 620, 540 and 80 bp were shown with the presence of a recognition site at one allele (+/−) while two amplicons at 540 and 80 bp were shown with the presence of recognition sites at both alleles (+/+). ^A^γ-H*ind*III, 5’ψβ-H*inc*II and 3’ψβ-H*inc*II were predominantly found with the absence of recognition sites at both alleles (−/−) as demonstrated in Table 2.

From the total 752 chromosomes, four different haplotypes were identified. Haplotype I (+ − − − −) was the predominant haplotype with a frequency of 0.9814 among 752 5’β-alleles. It was followed by Haplotype II (− + + − +), III (− + − + +) and IV (+ + + − +) with a frequency of 0.186. The distribution and frequency of β-globin gene cluster haplotypes are depicted in Table 3.

## Discussion

Sabah is located in East Malaysia in the northern region of Borneo Island, in which 60% of the total population is indigenous. The major ethnic groups are the Kadazandusun (25%), followed by the Bajau (15%), Murut (3%) and others. A high carrier rate of β-thalassaemia is reported in Sabah, with the Filipino β°-deletion as the predominant mutation (4).

The Filipino β°-deletion is an extensive deletion. Haplotyping can only be conducted at the 5’β-globin gene cluster, the region not affected by the deletion. Thus, the association between haplotype patterns at the 5’ and 3’β-globin gene cluster could not be determined. A previous study by Magaña (24) demonstrated poor linkage disequilibrium between the 5’β and 3’β-haplotype, as this region is very prone to recombination events. A higher recombination rate is found at the 3’β-globin gene cluster due to the sequences at the 3’β-region being predisposed to genetic events (24). Thus, the Filipino β°-deletion might be the consequence of genetic events occurring between the 5’ and 3’β-globin gene region during a recombination event.

In this study, 5’β-haplotype consisting of five RFLP markers could be assigned unequivocally for 752 of β-alleles [438 alleles from 219 β-TM patients and 314 alleles from 157 β-thalassaemia carriers]. The haplotype frequency estimation for all the β-TM patients and carriers with the Filipino β°-deletion from Sabah indicated Haplotype I as the predominant haplotype, followed by Haplotype II, III and IV. The Filipino β°-deletion was first reported in the Philippines (2). The close proximity of Sabah in North Borneo and the Philippines has allowed migration to occur, as demonstrated by the presence of the similar mutation of the Filipino β°-deletion (3). However, we are not able to determine the association between the populations in the Philippines as no haplotype information is currently available.

The presence of different haplotype patterns may indicate recombination events as well as a multicentric origin of the mutation (25). In this study, of the 752 β-alleles, a frequency of 0.9814 was found with Haplotype I (+ − − − −) and the remaining three haplotypes (Haplotypes II, III and IV) were reported at a low frequency (0.186), which is below a frequency of 0.02. This suggested a single origin in patients and carriers with the Filipino β°-deletion. This study demonstrates the Filipino β°-deletion in the indigenous population of Sabah is linked with a unique haplotype, Haplotype I. Our study implies that the Filipino β°-deletion with this haplotype might have arisen in the population with a common chromosomal background (25, 26).

Low genetic diversity was found in the Filipino β°-deletion and might be due to the founder effect, isolated living conditions and religious customs such as endogamous marriage with a high level of consanguinity, which is common in East Malaysia (27). The intermarriage rate was reported to be higher in East Malaysia (11.6%) compared to West Malaysia (3%) (27). This high rate of consanguineous marriages makes β-thalassaemia a huge health problem in this region.

The predominant Haplotype I found in the study subjects was also found in Austronesian Oceania (Micronesia, Melanesia and Polynesia) (28). Historically, the interior regions of Sabah, East Malaysia consisted of independent tribal societies. The earliest wave of human migration is believed to be Austronesian Mongoloids around 3000 BC, and represent the time when the indigenous people, such as the Murut and Kadazandusun, first arrived (29). This 5’β-haplotype was also encountered in the Han Chinese in Beijing, Xi’an, Kunming and other Asian populations such as the Khalkh, Buryat in Mongolia, Evenkis, Oroqen in North China, Japanese, Korean and Colombian Amerindian tribes (30). The haplotype in Sabah shows a similarity with these populations as the first modern human migrations were believed to be via mid-Asia, and probably settled in mainland Southeast Asia during the last Ice Age. Our study suggested 5’β-haplotype might not be sufficient in providing the full range of genetic information. SNP mapping, determination of haplotype patterns in mitochondria DNA (mtDNA), locus control region (LCR) repeat-sequencing and Y-chromosomes can provide additional genetic information (31–33).

## Conclusion

A predominant haplotype pattern was seen in β-thalassaemia major and carriers with the Filipino β°-deletion in Sabah. Haplotype I was seen in 98% of the study subjects. This is in keeping with a unicentric origin and an apparent single origin with low genetic diversity. This typical haplotype pattern has potential use as a marker for the Filipino β°-deletion.

## Figures and Tables

**Table 1:** Primer sequences and its product sizes, locations used for amplification of β-globin gene cluster

RFLP marker	Restriction enzymes	Primer	Product size	Absence (−)	Presence (+)	Position in GenBank:U01317	Reference
1	5’ε-H*inc*II	5’-TCT CTG TTT GAT GAC AAA TTC-3’ 5’-AGT CAT TGG TCA AGG CTG ACC-3’	760 bp	760 bp	446+314 bp	U18652-18672 U19391-19411	(19–21)
2	^G^γ-H*ind*III	5’-AGT GCT GCA AGA AGA ACA ACT ACC-3’ 5’-CTC TGC ATC ATG GGC AGT GAG CTC-3’	328 bp	328 bp	91+237 bp	U35677-35700 U35981-36004	(20, 21)
3	^A^γ-H*ind*III	5’-TGC TGC TAA TGC TTC ATT ACA A-3’ 5’-TAA ATG AGG AGC ATG CAC ACA C-3’	761 bp	761 bp	435+325 bp	U40358-40379 U41098-41119	(21)
4	5’ψβ-H*inc*II	5’-TCC TAT CCA TTA CTG TTC CTT GAA-3’ 5’-ATT GTC TTA TTC TAG AGA CGA TTT-3’	794 bp	794 bp	104+690 bp	U46686-46709 U47457-47480	(19, 21)
5	3’ψβ-H*inc*II	5’-TCT GCA TTT GAC TCT GTT AGC-3’ 5’-GGA CCC TAA CTG ATA TAA CTA-3’	620 bp	620 bp	540+80 bp	U49476-49496 U50069-50089	(19, 21, 22)

**Table 2:** Allele and genotype frequencies for five markers among 376 Filipino β°-deletion study subjects

Markers	RFLP markers	Population				

		Filipino β°-deletion (*n* = 376; 752 alleles)				

		Allelic frequencies		Genotype frequencies		
	
		+	−	+/+	+/−	−/−
1	5’ε-H*inc*II	739 (0.98)	13 (0.02)	363 (0.97)	13 (0.03)	0
2	^G^γ-H*ind*III	726 (0.97)	26 (0.03)	356 (0.94)	14 (0.04)	6 (0.02)
3	^A^γ-H*ind*III	8 (0.01)	744 (0.99)	0	8 (0.02)	368 (0.98)
4	5’ψβ-H*inc*II	6 (0.01)	746 (0.99)	0	6 (0.02)	370 (0.98)
5	3’ψβ-H*inc*II	14 (0.02)	738 (0.98)	0	14 (0.04)	362 (0.96)

**Table 3:** β-globin gene cluster haplotypes among 376 study subjects with homozygous and heterozygous Filipino β°-deletion

Haplotype	5’ to 3’*	Number of subjects *n* (%)	Cumulative frequency
I	+ − − − −	369 (98.1%)	0.9814
II	− + + − +	3 (0.8%)	0.9907
III	− + − + +	3 (0.8%)	0.9987
IV	+ + + − +	1 (0.3%)	1
Total		376	1

* The order of the sites from 5’ to 3’ is as follows:

5’H*inc*II to ε gene, H*ind*III to ^G^γ gene, H*ind*III to ^A^γ gene; H*inc*II to 5’ψβ gene and H*inc*II to 3’ψβ gene
